#  Hernia of Umbilical Cord associated with Cleft Lip and Palate, and Congenital Glaucoma 

**Published:** 2015-10-01

**Authors:** Irom Keshorjit Singh

**Affiliations:** Department of Surgery (Pediatric Surgery), Regional Institute of Medical Sciences, Imphal, 795004, Manipur.

**Dear Sir**

Congenital herniation of the umbilical cord (CHUC) is a rare anomaly due to incomplete closure of umbilical ring and is different from abdominal wall defects. Intestinal atresias have been reported as a common associated anomaly. We herein present two cases hernia of umbilical cord associated with non gastro-intestinal anomalies. In one neonate, cleft lip and palate, PVID along with G6PD deficiency and in another neonate congenital glaucoma along with Meckel’s diverticulum were found as associated anomalies.

Case 1: A full term male newborn (product of non-consanguineous marriage) presented with complete cleft lip and palate of the left side with a swelling at the base of the umbilical cord. There was no anterior abdominal wall defect. The umbilical ring was complete and covered by a cuff of skin. At the fundus of the sac, there was an orifice from which the meconium was discharged suggestive of a CHUC with patent vitellointestinal duct (PVID) (Fig. 1). On exploration, a loop of terminal ileum along with the PVID was found in the CHUC. The sac along with the PVID (primary ileal repair done) was excised and defect closed followed by umbilicoplasty. Postoperatively, the bilirubin level increased to 20 mg/dl on day 3 for which phototherapy was initiated. Since there was prolonged hyperbilirubinemia, Glucose-6-phosphate dehydrogenase (G6PD) screening was done which confirmed its deficiency. Baby was discharged after 7 days with proper advice of feeding as there was cleft lip and palate. The baby returned to the hospital with aspiration pneumonia after 15 days and succumbed to it.

**Figure:**
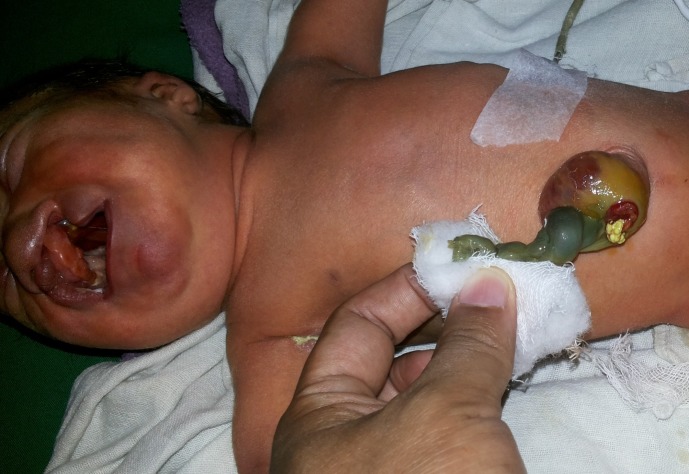
Figure 1: Cleft lip and palate on left, orifice on the sac (PVID) of CHUC with stool.

Case 2: A term male baby was born to a 30 years old woman of non consanguineous marriage. There were no perinatal events. On examination there was CHUC. The ophthalmic surgeon examined for corneal enlargement (corneal diameter of 15 mm), corneal clouding/opacity and diagnosed as primary congenital glaucoma with intraocular pressure of 28 mm of Hg (Fig. 2). On exploration, the umbilical ring was found patent along with a Meckel’s diverticulum (Fig.2). Diverticulectomy was done and defect closed by umbilicoplasty. Post operative recovery was uneventful. The baby is on follow-up of ophthalmic surgeon for glaucoma.

**Figure:**
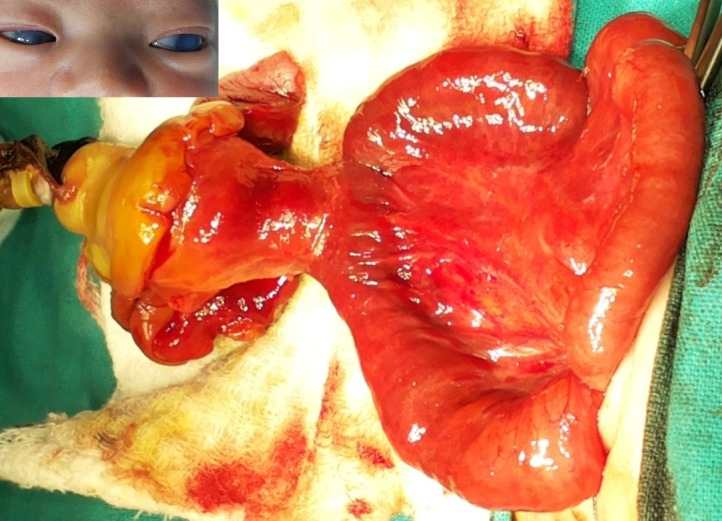
Figure 2: Dissected sac with the Meckel’s diverticulum inside the sac of CHUC. Inset showed enlarged cornea with corneal clouding.

Umbilical cord is a vital link of the developing fetus through which all the nourishments occur and the developing bowels transit through the umbilical ring in the first trimester [1]. Multiple anomalies can occur at umbilicus which may be associated with local as well as global developmental anomalies. CHUC is regarded as a minor defect in obliteration of umbilicus and most of the reported complications are due to herniation of gut through the small defect. There have been case reports of CHUC associated with other gastrointestinal anomalies such as intestinal atresia, Meckel’s diverticulum, congenital short gut, intestinal perforation, PVID, persistent cloaca etc. [1-6] In our first case, there was a PVID along with the herniation of the cord. The associated cleft lip and palate along with the G6PD deficiency has not been reported earlier. The second case was associated with primary congenital glaucoma which is also the first case of such association. Early detection and initiation of treatment is needed to avoid blindness. 

To conclude, there could be associated anomalies in other systems apart from gastro-intestinal system in cases of congenital herniation of the umbilical cord. So, a detailed examination is necessary to detect these associated anomalies in order to initiate timely treatment.

## Footnotes

**Source of Support:** Nil

**Conflict of Interest:** None
